# *catena*-Poly[[[bis­(4-bromo­benzoato-κ*O*)zinc]-μ-1,2-bis­(4-pyrid­yl)ethene-κ^2^ *N*:*N*′] acetonitrile monosolvate]

**DOI:** 10.1107/S1600536811042188

**Published:** 2011-10-22

**Authors:** Ni-Ya Li, Deng-Ming Sun

**Affiliations:** a College of Chemistry and Materials Science, Huaibei Normal University, Huaibei 235000, Anhui, People’s Republic of China

## Abstract

In the title coordination compound, {[Zn(C_7_H_4_BrO_2_)_2_(C_12_H_10_N_2_)]·CH_3_CN}_*n*_, the Zn^II^ atom is four-coordinated in a distorted tetra­hedral environment by two carboxyl­ate O atoms from two different 4-bromo­benzoate (bpe) ligands and two N atoms from two symmetry-related 1,2-bis­(4-pyrid­yl)ethene ligands. The Zn^II^ atoms are bridged by the bpe ligands, which lie across centres of inversion, forming a zigzag chain along [001]. The void space of each unit cell is occupied by an acetonitrile solvent mol­ecule, which is connected to the complex mol­ecule by a weak C—H⋯N hydrogen bond.

## Related literature

For zigzag chains constructed by Zn^II^, mono-carboxyl­ate ligands and dipyridyl ligands, see: Gao *et al.* (2010 ▶); Kwak *et al.* (2009 ▶); Ng *et al.* (2004 ▶). 
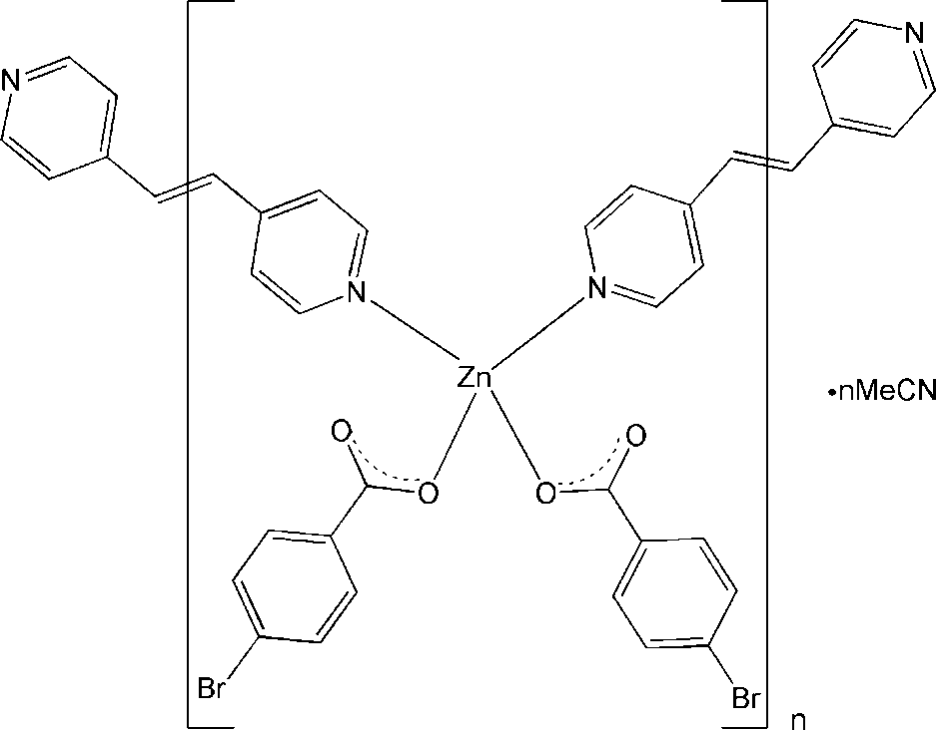


## Experimental

### 

#### Crystal data

[Zn(C_7_H_4_BrO_2_)_2_(C_12_H_10_N_2_)]·C_2_H_3_N
*M* *_r_* = 688.67
Triclinic, 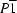

*a* = 6.2738 (13) Å
*b* = 11.852 (2) Å
*c* = 19.496 (4) Å
α = 105.25 (3)°
β = 94.63 (3)°
γ = 99.87 (3)°
*V* = 1365.8 (5) Å^3^
*Z* = 2
Mo *K*α radiation
μ = 3.86 mm^−1^
*T* = 223 K
0.20 × 0.10 × 0.10 mm

#### Data collection

Rigaku MercuryCCD area-detector diffractometer
Absorption correction: multi-scan (*REQAB*; Jacobson, 1998 ▶) *T*_min_ = 0.512, *T*_max_ = 0.699
13203 measured reflections
6152 independent reflections
3934 reflections with *I* > 2σ(*I*)
*R*_int_ = 0.045

#### Refinement

*R*[*F* ^2^ > 2σ(*F* ^2^)] = 0.053
*wR*(*F* ^2^) = 0.110
*S* = 1.05
6152 reflections
344 parameters
H-atom parameters constrained
Δρ_max_ = 0.51 e Å^−3^
Δρ_min_ = −0.54 e Å^−3^

### 

Data collection: *CrystalClear* (Rigaku, 2001 ▶); cell refinement: *CrystalClear*; data reduction: *CrystalStructure* (Rigaku/MSC, 2004 ▶); program(s) used to solve structure: *SHELXTL* (Sheldrick, 2008 ▶); program(s) used to refine structure: *SHELXTL*; molecular graphics: *SHELXTL*; software used to prepare material for publication: *SHELXTL* and *PLATON* (Spek, 2009 ▶).

## Supplementary Material

Crystal structure: contains datablock(s) I, global. DOI: 10.1107/S1600536811042188/bq2311sup1.cif

Structure factors: contains datablock(s) I. DOI: 10.1107/S1600536811042188/bq2311Isup2.hkl

Additional supplementary materials:  crystallographic information; 3D view; checkCIF report

## Figures and Tables

**Table 1:** Hydrogen-bond geometry (Å, °)

*D*—H⋯*A*	*D*—H	H⋯*A*	*D*⋯*A*	*D*—H⋯*A*
C12—H12⋯N3	0.94	2.53	3.436 (8)	162

**Fig. 1.**
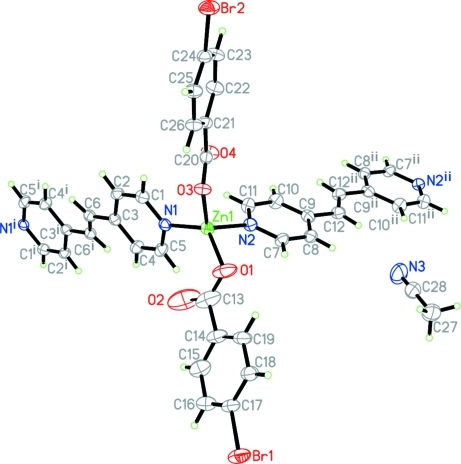
Coordination environment of ZnII atom in the compound with nonhydrogen atoms represented by thermal ellipsoids draw at 30% probability level. [Symmetry codes, i: - x - 1, - y + 1, - z + 1; ii: - x, - y + 2, - z + 2.]

**Fig. 2.**
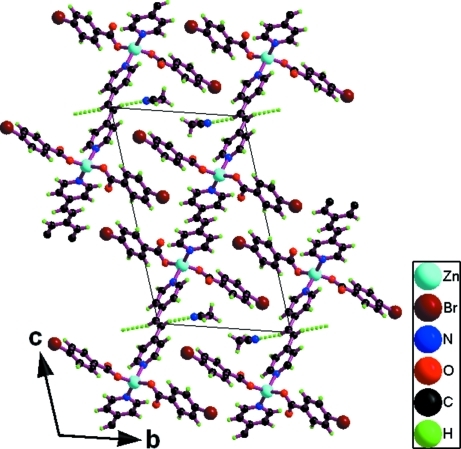
View of the unit-cell contents of the title compound showing weak intermolecular H-bond with dashed lines.

## supplementary crystallographic information

### Comment

In recent years, a large number of coordination polymers assembled from carboxylates and pyridyl-like ligands have been extensively investigated. Among these coordination polymers, most of them are constructed by polycarboxylates and dipyridyl ligands, the complexes assembled from mono-carboxylate and dipyridyl ligands are still rare.

In this work, 4-bromobenzoate (BBA) and 1,2-bis(4-pyridyl)ethene (bpe) were employed to react with Zn^II^ and thus afford the title complex, {[Zn(C_7_H_4_O_2_Br)_2_(C_12_H_10_N_2_)].(C_2_H_3_N)}_n_ (I). In (I), the Zn^II^ atom lies on a twofold rotation axis that relates one BBA ligand to the other as well as one bpe ligand to the other; the coordination geometry is a distorted tetrahedron (Fig. 1). The Zn—O (1.958 (3) – 1.985 (4) Å) and Zn—N (2.049 (3) – 2.060 (3) Å) bond lengths are comparable to those reported for similar complexes (Gao *et al.*, 2010; Kwak *et al.*, 2009; Ng *et al.*, 2004). The Zn^II^ centres are linked by the bpe ligands to form a one-dimensional zigzag chain (Fig. 2). A non-coordinated solvent molecule of acetonitrile occupies the interstitial voids within the unit cell. It is noted that there is a weak C12—H12···N3 (Table 1) intermolecular hydrogen bond in the structure (Fig. 2). This weak interaction connects the main molecule with the solvent molecule.

### Experimental

To a 25 ml Teflon-lined stainless steel autoclave was loaded Zn(NO_3_)_2_.6H_2_O (149 mg, 0.5 mmol), 4-bromobenzoic acid (200 mg, 1 mmol), 1,2-bis(4-pyridyl)ethene (91 mg, 0.5 mmol), H_2_O (8 ml) and acetonitrile (8 ml). The autoclave was sealed and heated in an oven to 423 K for three days, and then cooled to ambient temperature at the rate of 5 K/h to form yellow crystals of (I). Yield: 238 mg (69% yield based on Zn). Anal. calcd. for C_28_H_21_Br_2_N_3_O_4_Zn: C, 48.83; H, 3.07; N, 6.10. Found: C, 50.05; H, 3.22; N, 6.37.

### Refinement

The C-bound H atoms were positioned geometrically, with C–H = 0.97 Å (methyl) or 0.94 Å (phenyl, pyridyl and vinyl), and refined as riding, with *U*_iso_(H) = 1.5*U*_eq_(C) for methyl groups or 1.2*U*_eq_(C) otherwise.

### Crystal data

**Table:**

[Zn(C_7_H_4_BrO_2_)_2_(C_12_H_10_N_2_)]·C_2_H_3_N	*Z* = 2
*M**_r_* = 688.67	*F*(000) = 684
Triclinic, *P*1	*D*_x_ = 1.675 Mg m^−^^3^
Hall symbol: -P 1	Mo *K*α radiation, λ = 0.71073 Å
*a* = 6.2738 (13) Å	Cell parameters from 5974 reflections
*b* = 11.852 (2) Å	θ = 3.2–27.5°
*c* = 19.496 (4) Å	µ = 3.86 mm^−^^1^
α = 105.25 (3)°	*T* = 223 K
β = 94.63 (3)°	Block, yellow
γ = 99.87 (3)°	0.20 × 0.10 × 0.10 mm
*V* = 1365.8 (5) Å^3^	

### Data collection

**Table:**

Rigaku MercuryCCD area-detector diffractometer	6152 independent reflections
Radiation source: fine-focus sealed tube	3934 reflections with *I* > 2σ(*I*)
graphite	*R*_int_ = 0.045
ω scans	θ_max_ = 27.5°, θ_min_ = 3.2°
Absorption correction: multi-scan (REQAB; Jacobson, 1998)	*h* = −7→8
*T*_min_ = 0.512, *T*_max_ = 0.699	*k* = −15→15
13203 measured reflections	*l* = −25→21

### Refinement

**Table:**

Refinement on *F*^2^	Primary atom site location: structure-invariant direct methods
Least-squares matrix: full	Secondary atom site location: difference Fourier map
*R*[*F*^2^ > 2σ(*F*^2^)] = 0.053	Hydrogen site location: inferred from neighbouring sites
*wR*(*F*^2^) = 0.110	H-atom parameters constrained
*S* = 1.05	*w* = 1/[σ^2^(*F*_o_^2^) + (0.0378*P*)^2^] where *P* = (*F*_o_^2^ + 2*F*_c_^2^)/3
6152 reflections	(Δ/σ)_max_ = 0.001
344 parameters	Δρ_max_ = 0.51 e Å^−^^3^
0 restraints	Δρ_min_ = −0.54 e Å^−^^3^

### Special details

**Table:**

Geometry. All e.s.d.'s (except the e.s.d. in the dihedral angle between two l.s. planes) are estimated using the full covariance matrix. The cell e.s.d.'s are taken into account individually in the estimation of e.s.d.'s in distances, angles and torsion angles; correlations between e.s.d.'s in cell parameters are only used when they are defined by crystal symmetry. An approximate (isotropic) treatment of cell e.s.d.'s is used for estimating e.s.d.'s involving l.s. planes.
Refinement. Refinement of *F*^2^ against ALL reflections. The weighted *R*-factor *wR* and goodness of fit *S* are based on *F*^2^, conventional *R*-factors *R* are based on *F*, with *F* set to zero for negative *F*^2^. The threshold expression of *F*^2^ > σ(*F*^2^) is used only for calculating *R*-factors(gt) *etc*. and is not relevant to the choice of reflections for refinement. *R*-factors based on *F*^2^ are statistically about twice as large as those based on *F*, and *R*- factors based on ALL data will be even larger.

### Fractional atomic coordinates and isotropic or equivalent isotropic displacement parameters (Å^2^)

**Table:**

	*x*	*y*	*z*	*U*_iso_*/*U*_eq_	
Zn1	0.44987 (7)	0.71792 (4)	0.71734 (3)	0.03921 (15)	
Br1	1.37712 (10)	1.24685 (5)	0.59537 (3)	0.0756 (2)	
Br2	0.94349 (9)	0.17043 (4)	0.87084 (3)	0.06577 (18)	
N1	0.1772 (5)	0.6343 (3)	0.64483 (16)	0.0374 (8)	
N2	0.3188 (5)	0.8162 (3)	0.80109 (18)	0.0401 (8)	
O1	0.6984 (7)	0.8429 (3)	0.7125 (3)	0.0948 (15)	
O2	0.4838 (8)	0.8466 (5)	0.6190 (4)	0.148 (3)	
O3	0.6068 (4)	0.5980 (2)	0.73677 (15)	0.0438 (7)	
O4	0.3088 (5)	0.5406 (3)	0.78312 (16)	0.0513 (8)	
C1	0.1024 (7)	0.5156 (4)	0.6262 (2)	0.0431 (10)	
H1	0.1853	0.4677	0.6438	0.052*	
C2	−0.0907 (7)	0.4620 (4)	0.5825 (2)	0.0412 (10)	
H2	−0.1379	0.3788	0.5711	0.049*	
C3	−0.2171 (6)	0.5292 (3)	0.5550 (2)	0.0387 (10)	
C4	−0.1384 (7)	0.6510 (4)	0.5738 (2)	0.0449 (11)	
H4	−0.2182	0.7006	0.5567	0.054*	
C5	0.0578 (7)	0.6997 (4)	0.6177 (2)	0.0455 (11)	
H5	0.1099	0.7825	0.6291	0.055*	
C6	−0.4231 (6)	0.4716 (4)	0.5084 (2)	0.0398 (10)	
H6	−0.4489	0.3883	0.4887	0.048*	
C7	0.4393 (7)	0.9177 (3)	0.8455 (2)	0.0448 (11)	
H7	0.5797	0.9455	0.8355	0.054*	
C8	0.3661 (7)	0.9821 (4)	0.9046 (2)	0.0445 (11)	
H8	0.4549	1.0537	0.9334	0.053*	
C9	0.1640 (7)	0.9436 (4)	0.9224 (2)	0.0419 (10)	
C10	0.0358 (7)	0.8385 (4)	0.8758 (2)	0.0500 (11)	
H10	−0.1054	0.8095	0.8846	0.060*	
C11	0.1194 (7)	0.7783 (4)	0.8169 (2)	0.0495 (11)	
H11	0.0329	0.7075	0.7863	0.059*	
C12	0.0908 (7)	1.0147 (4)	0.9876 (2)	0.0475 (11)	
H12	0.1836	1.0872	1.0132	0.057*	
C13	0.6547 (13)	0.8825 (6)	0.6615 (5)	0.094 (2)	
C14	0.8324 (9)	0.9765 (4)	0.6480 (3)	0.0626 (15)	
C15	0.7939 (9)	1.0245 (5)	0.5934 (3)	0.0739 (16)	
H15	0.6559	1.0015	0.5656	0.089*	
C16	0.9529 (8)	1.1066 (4)	0.5776 (3)	0.0633 (14)	
H16	0.9235	1.1401	0.5401	0.076*	
C17	1.1540 (8)	1.1382 (4)	0.6178 (3)	0.0521 (12)	
C18	1.1993 (8)	1.0931 (4)	0.6739 (3)	0.0566 (12)	
H18	1.3377	1.1165	0.7015	0.068*	
C19	1.0344 (9)	1.0115 (4)	0.6889 (3)	0.0620 (14)	
H19	1.0615	0.9800	0.7275	0.074*	
C20	0.4967 (7)	0.5330 (3)	0.7693 (2)	0.0388 (10)	
C21	0.6034 (6)	0.4408 (3)	0.7914 (2)	0.0381 (9)	
C22	0.5102 (7)	0.3804 (4)	0.8367 (2)	0.0505 (11)	
H22	0.3778	0.3959	0.8525	0.061*	
C23	0.6067 (7)	0.2979 (4)	0.8591 (2)	0.0529 (12)	
H23	0.5431	0.2579	0.8904	0.063*	
C24	0.7997 (7)	0.2758 (3)	0.8343 (2)	0.0439 (10)	
C25	0.8937 (7)	0.3311 (4)	0.7881 (2)	0.0484 (11)	
H25	1.0233	0.3130	0.7710	0.058*	
C26	0.7951 (7)	0.4148 (4)	0.7667 (2)	0.0451 (10)	
H26	0.8590	0.4541	0.7351	0.054*	
C27	0.8391 (11)	1.4073 (6)	1.0550 (3)	0.099 (2)	
H27A	0.8972	1.4411	1.1053	0.148*	
H27B	0.8351	1.4712	1.0327	0.148*	
H27C	0.9315	1.3557	1.0313	0.148*	
C28	0.6195 (13)	1.3385 (6)	1.0485 (3)	0.092 (2)	
N3	0.4488 (11)	1.2851 (5)	1.0428 (3)	0.121 (2)	

### Atomic displacement parameters (Å^2^)

**Table:**

	*U*^11^	*U*^22^	*U*^33^	*U*^12^	*U*^13^	*U*^23^
Zn1	0.0397 (3)	0.0422 (3)	0.0404 (3)	0.0136 (2)	0.0121 (2)	0.0142 (2)
Br1	0.0754 (4)	0.0562 (3)	0.1030 (5)	0.0027 (3)	0.0328 (3)	0.0365 (3)
Br2	0.0704 (4)	0.0566 (3)	0.0812 (4)	0.0200 (3)	0.0006 (3)	0.0365 (3)
N1	0.046 (2)	0.0385 (18)	0.0321 (18)	0.0153 (17)	0.0117 (16)	0.0110 (15)
N2	0.0378 (19)	0.0420 (19)	0.043 (2)	0.0104 (17)	0.0123 (16)	0.0129 (16)
O1	0.129 (4)	0.051 (2)	0.130 (4)	0.029 (2)	0.092 (3)	0.041 (2)
O2	0.072 (3)	0.126 (4)	0.265 (8)	−0.003 (3)	0.060 (4)	0.093 (5)
O3	0.0402 (16)	0.0477 (17)	0.0550 (18)	0.0112 (14)	0.0136 (14)	0.0302 (15)
O4	0.0408 (17)	0.064 (2)	0.063 (2)	0.0195 (15)	0.0179 (15)	0.0325 (16)
C1	0.045 (2)	0.046 (3)	0.044 (2)	0.018 (2)	0.015 (2)	0.016 (2)
C2	0.046 (2)	0.036 (2)	0.044 (2)	0.008 (2)	0.015 (2)	0.0129 (19)
C3	0.041 (2)	0.042 (2)	0.036 (2)	0.009 (2)	0.0153 (19)	0.0131 (19)
C4	0.045 (2)	0.041 (2)	0.051 (3)	0.015 (2)	0.006 (2)	0.012 (2)
C5	0.050 (3)	0.035 (2)	0.051 (3)	0.015 (2)	0.012 (2)	0.006 (2)
C6	0.041 (2)	0.042 (2)	0.036 (2)	0.0057 (19)	0.0120 (19)	0.0109 (18)
C7	0.044 (2)	0.038 (2)	0.051 (3)	0.004 (2)	0.014 (2)	0.010 (2)
C8	0.054 (3)	0.038 (2)	0.043 (2)	0.014 (2)	0.012 (2)	0.0110 (19)
C9	0.046 (3)	0.047 (2)	0.041 (2)	0.022 (2)	0.011 (2)	0.016 (2)
C10	0.036 (2)	0.056 (3)	0.059 (3)	0.012 (2)	0.019 (2)	0.012 (2)
C11	0.044 (3)	0.051 (3)	0.052 (3)	0.012 (2)	0.008 (2)	0.009 (2)
C12	0.052 (3)	0.042 (2)	0.052 (3)	0.012 (2)	0.012 (2)	0.017 (2)
C13	0.091 (5)	0.056 (4)	0.156 (7)	0.031 (4)	0.083 (5)	0.037 (4)
C14	0.064 (3)	0.043 (3)	0.097 (4)	0.021 (3)	0.050 (3)	0.030 (3)
C15	0.053 (3)	0.066 (3)	0.108 (5)	0.013 (3)	0.021 (3)	0.030 (3)
C16	0.063 (3)	0.060 (3)	0.080 (4)	0.017 (3)	0.017 (3)	0.037 (3)
C17	0.056 (3)	0.041 (2)	0.070 (3)	0.013 (2)	0.027 (3)	0.027 (2)
C18	0.065 (3)	0.047 (3)	0.062 (3)	0.012 (2)	0.017 (3)	0.021 (2)
C19	0.088 (4)	0.046 (3)	0.068 (3)	0.023 (3)	0.038 (3)	0.030 (3)
C20	0.041 (2)	0.037 (2)	0.036 (2)	0.0059 (19)	0.0045 (19)	0.0066 (19)
C21	0.033 (2)	0.041 (2)	0.041 (2)	0.0015 (18)	0.0040 (18)	0.0168 (19)
C22	0.039 (2)	0.064 (3)	0.058 (3)	0.012 (2)	0.018 (2)	0.030 (2)
C23	0.053 (3)	0.061 (3)	0.057 (3)	0.012 (2)	0.014 (2)	0.037 (2)
C24	0.046 (3)	0.038 (2)	0.050 (3)	0.007 (2)	−0.003 (2)	0.018 (2)
C25	0.041 (2)	0.051 (3)	0.059 (3)	0.009 (2)	0.012 (2)	0.025 (2)
C26	0.043 (2)	0.045 (2)	0.055 (3)	0.008 (2)	0.014 (2)	0.026 (2)
C27	0.115 (5)	0.091 (4)	0.079 (4)	−0.020 (4)	0.035 (4)	0.023 (3)
C28	0.127 (6)	0.076 (4)	0.057 (4)	−0.017 (4)	0.019 (4)	0.014 (3)
N3	0.120 (5)	0.114 (5)	0.089 (4)	−0.053 (4)	0.012 (4)	0.011 (3)

### Geometric parameters (Å, °)

**Table:**

Zn1—O3	1.958 (3)	C10—H10	0.9400
Zn1—O1	1.985 (4)	C11—H11	0.9400
Zn1—N1	2.049 (3)	C12—C12^ii^	1.302 (8)
Zn1—N2	2.060 (3)	C12—H12	0.9400
Br1—C17	1.894 (5)	C13—C14	1.526 (8)
Br2—C24	1.906 (4)	C14—C15	1.357 (7)
N1—C5	1.338 (5)	C14—C19	1.377 (7)
N1—C1	1.345 (5)	C15—C16	1.382 (7)
N2—C7	1.342 (5)	C15—H15	0.9400
N2—C11	1.343 (5)	C16—C17	1.367 (7)
O1—C13	1.236 (9)	C16—H16	0.9400
O2—C13	1.238 (9)	C17—C18	1.367 (7)
O3—C20	1.272 (5)	C18—C19	1.396 (7)
O4—C20	1.243 (5)	C18—H18	0.9400
C1—C2	1.372 (6)	C19—H19	0.9400
C1—H1	0.9400	C20—C21	1.510 (5)
C2—C3	1.386 (5)	C21—C22	1.380 (6)
C2—H2	0.9400	C21—C26	1.384 (5)
C3—C4	1.382 (5)	C22—C23	1.378 (6)
C3—C6	1.464 (5)	C22—H22	0.9400
C4—C5	1.382 (6)	C23—C24	1.381 (6)
C4—H4	0.9400	C23—H23	0.9400
C5—H5	0.9400	C24—C25	1.360 (6)
C6—C6^i^	1.333 (7)	C25—C26	1.389 (6)
C6—H6	0.9400	C25—H25	0.9400
C7—C8	1.365 (5)	C26—H26	0.9400
C7—H7	0.9400	C27—C28	1.453 (9)
C8—C9	1.375 (6)	C27—H27A	0.9700
C8—H8	0.9400	C27—H27B	0.9700
C9—C10	1.403 (6)	C27—H27C	0.9700
C9—C12	1.484 (6)	C28—N3	1.128 (8)
C10—C11	1.377 (6)		
			
O3—Zn1—O1	100.35 (16)	O1—C13—O2	124.7 (7)
O3—Zn1—N1	109.30 (12)	O1—C13—C14	116.8 (8)
O1—Zn1—N1	129.94 (19)	O2—C13—C14	118.4 (8)
O3—Zn1—N2	117.36 (13)	C15—C14—C19	118.9 (5)
O1—Zn1—N2	98.90 (14)	C15—C14—C13	119.7 (7)
N1—Zn1—N2	101.82 (13)	C19—C14—C13	121.3 (6)
C5—N1—C1	117.4 (3)	C14—C15—C16	121.6 (5)
C5—N1—Zn1	119.6 (3)	C14—C15—H15	119.2
C1—N1—Zn1	122.9 (3)	C16—C15—H15	119.2
C7—N2—C11	117.4 (4)	C17—C16—C15	118.6 (5)
C7—N2—Zn1	120.1 (3)	C17—C16—H16	120.7
C11—N2—Zn1	122.3 (3)	C15—C16—H16	120.7
C13—O1—Zn1	109.7 (5)	C18—C17—C16	121.8 (5)
C20—O3—Zn1	111.2 (2)	C18—C17—Br1	118.8 (4)
N1—C1—C2	122.2 (4)	C16—C17—Br1	119.4 (4)
N1—C1—H1	118.9	C17—C18—C19	118.1 (5)
C2—C1—H1	118.9	C17—C18—H18	120.9
C1—C2—C3	120.8 (4)	C19—C18—H18	120.9
C1—C2—H2	119.6	C14—C19—C18	120.9 (5)
C3—C2—H2	119.6	C14—C19—H19	119.6
C4—C3—C2	116.7 (4)	C18—C19—H19	119.6
C4—C3—C6	122.7 (4)	O4—C20—O3	124.0 (4)
C2—C3—C6	120.6 (4)	O4—C20—C21	119.0 (4)
C5—C4—C3	119.8 (4)	O3—C20—C21	117.0 (3)
C5—C4—H4	120.1	C22—C21—C26	118.7 (4)
C3—C4—H4	120.1	C22—C21—C20	120.3 (4)
N1—C5—C4	123.0 (4)	C26—C21—C20	121.0 (4)
N1—C5—H5	118.5	C23—C22—C21	121.6 (4)
C4—C5—H5	118.5	C23—C22—H22	119.2
C6^i^—C6—C3	124.9 (5)	C21—C22—H22	119.2
C6^i^—C6—H6	117.5	C22—C23—C24	118.0 (4)
C3—C6—H6	117.5	C22—C23—H23	121.0
N2—C7—C8	122.6 (4)	C24—C23—H23	121.0
N2—C7—H7	118.7	C25—C24—C23	122.2 (4)
C8—C7—H7	118.7	C25—C24—Br2	119.9 (3)
C7—C8—C9	120.8 (4)	C23—C24—Br2	117.9 (4)
C7—C8—H8	119.6	C24—C25—C26	118.9 (4)
C9—C8—H8	119.6	C24—C25—H25	120.6
C8—C9—C10	117.0 (4)	C26—C25—H25	120.6
C8—C9—C12	119.5 (4)	C21—C26—C25	120.5 (4)
C10—C9—C12	123.5 (4)	C21—C26—H26	119.7
C11—C10—C9	119.1 (4)	C25—C26—H26	119.7
C11—C10—H10	120.5	C28—C27—H27A	109.5
C9—C10—H10	120.5	C28—C27—H27B	109.5
N2—C11—C10	123.1 (4)	H27A—C27—H27B	109.5
N2—C11—H11	118.5	C28—C27—H27C	109.5
C10—C11—H11	118.5	H27A—C27—H27C	109.5
C12^ii^—C12—C9	125.7 (6)	H27B—C27—H27C	109.5
C12^ii^—C12—H12	117.2	N3—C28—C27	179.3 (7)
C9—C12—H12	117.2		

Symmetry codes: (i) −*x*−1, −*y*+1, −*z*+1; (ii) −*x*, −*y*+2, −*z*+2.

### Hydrogen-bond geometry (Å, °)

**Table:**

*D*—H···*A*	*D*—H	H···*A*	*D*···*A*	*D*—H···*A*
C12—H12···N3	0.94	2.53	3.436 (8)	162.
